# Illness Meaning-Making in an Oncology Support Group: A Longitudinal Lexical Correspondence Analysis of Group Discourse

**DOI:** 10.3390/ejihpe16100159

**Published:** 2026-10-07

**Authors:** Claudia Venuleo, Paolo Colavero, Silvia De Padova, Monica Scotto Di Carlo

**Affiliations:** 1 Department of Human and Social Sciences, University of Salento, 73100 Lecce, Italy; paolo.colavero@unisalento.it; 2 Hospital Psychology Unit, Vito Fazzi Hospital, Local Health Authority (ASL) of Lecce, 73100 Lecce, Italy; silvia.depadova@asl.lecce.it (S.D.P.); monica.scottodicarlo@asl.lecce.it (M.S.D.C.)

**Keywords:** cancer, meaning-making, support groups, illness experience, group discourse, psychosocial care, longitudinal research

## Abstract

Living with cancer involves an ongoing effort to make sense of experiences that disrupt identity, relationships, and future expectations. Meaning-making is embedded in interpersonal and healthcare contexts, including psychosocial support settings where illness experiences are shared with others. This exploratory longitudinal study examined the evolving configuration of illness meanings in an oncology support group. Verbatim transcripts from 19 sessions involving 11 women with recent or ongoing cancer treatment were analyzed using Lexical Correspondence Analysis across four temporal blocks, with speaker roles as illustrative variables. Descriptive analyses of group climate, working alliance, and open-ended responses contextualized discursive findings. Two semantic dimensions organized group discourse: one contrasting *illness as situated experience* with *experiential elaboration*, and the other *emotional elaboration* with *embodied experiences of safety*. After Benzécri’s adjustment, the two dimensions accounted for 33.86% of the explained inertia. Their salience varied across temporal blocks rather than linearly. Illness-related meanings remained salient while becoming embedded within experiential, relational, and embodied configurations. Group process data indicated engagement, low conflict, and collaboration, while open-ended responses highlighted normalization, belonging, emotional safety, emotional resonance, coping resources, and facilitator support. The study contributes to process-oriented psycho-oncology research by examining how illness meanings are negotiated within a support-group.

## 1. Introduction

Living with cancer entails disruptions that extend beyond the biological sphere, challenging taken-for-granted assumptions about identity, relationships, and future life trajectories (Almeida et al., 2022; Bülow & Hydén, 2003; Frank, 1995; Hulse et al., 2024). Adapting to cancer therefore involves more than managing symptoms and treatment demands; it also requires an ongoing effort to make sense of experiences that may unsettle previously established meanings about the self, others, and everyday life (Rodin et al., 2025).

Making sense of cancer, however, is not an exclusively individual process. The meanings people develop around illness are embedded in the relational, social, and healthcare contexts in which cancer is experienced. Support from significant others and social networks, as well as interactions with healthcare professionals and care settings, contribute to the interpersonal and institutional context within which illness experiences are articulated and given meaning (Bottaro et al., 2023; Venuleo et al., 2026b). Healthcare settings may also provide structured psychosocial resources, such as psychological support and professionally facilitated group interventions, that complement medical treatment by addressing the emotional, relational, and adjustment-related challenges associated with cancer. Evidence from reviews of psychosocial and supportive interventions indicates potential benefits for psychological well-being, psychological adaptation, and quality of life among people living with cancer (Lingens et al., 2021; Peixoto et al., 2021). Qualitative studies have further examined how patients experience and make use of group-based interventions. Brandão et al. (2019), for example, found that women participating in supportive-expressive group therapy identified the expression and normalization of emotions, increased social support, and learning through sharing experiences as particularly helpful aspects of the group. More recently, Iannopollo et al. (2024), examining a narrative psychotherapy pathway for cancer patients, described group narration as a process of relational co-construction of meaning and reported changes in participants’ self-narratives and ways of making sense of the illness experience.

Within research on psychological adaptation to cancer and other life-threatening illnesses, meaning-making has long been recognized as a central process, with substantial evidence linking it to emotional adjustment, psychological well-being, and adaptation outcomes (Almeida et al., 2022; Martin et al., 2026; Quinto et al., 2022; Park & Hanna, 2022; Sun et al., 2024; Visser et al., 2024). While early approaches emphasized the restoration of coherence following biographical disruption (Bury, 1982), constructivist and narrative perspectives have highlighted meaning-making as a process of reconstructing identity and continuity after disruptive life events (Neimeyer, 2001, 2006). More recent perspectives have further expanded this view by conceptualizing meaning-making as an ongoing, relational and situated process through which meanings are dynamically constituted in experience and interaction with others (De Luca Picione et al., 2017; Hartog et al., 2020; Lemmo et al., 2025; Martino et al., 2019, 2023a; Ocampo-Ocampo et al., 2026).

Within a semiotic-cultural and dialogical perspective, this relationality is understood in terms of the dynamic organization of meanings within communicative exchanges. Meanings are not considered fixed contents that individuals bring into interaction; rather, they emerge through evolving relations among signs, as participants respond to, elaborate, and transform one another’s contributions within situated practices of illness and care (Salvatore et al., 2024; Venuleo et al., 2018, 2026b). This view has important implications for the study of meaning-making. If meanings are dynamically constructed within communicative exchanges, discourse cannot be treated merely as a vehicle through which pre-existing meanings are expressed. Rather, discourse provides the empirical field in which meanings can be examined as they are negotiated and reconfigured through interaction (Linell, 2009, 2017).

This perspective is particularly relevant to oncology support groups, where illness experiences are shared within an ongoing interpersonal context (Arizu-Onassis et al., 2025; Foulkes, 1964; Iannopollo et al., 2024; Li et al., 2025; Yalom & Leszcz, 2020). Thus, groups represent valuable settings for examining meaning-making as an unfolding relational process rather than solely as an individual outcome. Although evidence suggests that support groups and peer-based interventions can improve psychological and psychosocial outcomes among people living with cancer (Kiemen et al., 2023; Li et al., 2025), less is known about the processes through which these benefits may emerge. Existing research has examined illness narratives, therapeutic processes, and psychosocial outcomes, providing evidence on how participants experience group interventions and how illness-related meanings are explored and elaborated within them, but comparatively little attention has been paid to how illness meanings are collectively negotiated and transformed through group interaction over time. Professionally facilitated support groups provide a setting in which individual illness experiences are brought into contact with those of others, creating an interpersonal space where meanings can be shared, negotiated, and reconfigured. Examining how discourse changes within such settings may therefore contribute to understanding how interpersonal and healthcare contexts shape the psychological experience of cancer.

From a semiotic-cultural perspective, meanings are not reducible to recurring thematic contents but emerge from the relations among signs within communicative exchanges (Linell, 2009; Salvatore & Freda, 2011). Consequently, understanding collective meaning-making requires attention to the semantic organization of discourse, rather than an exclusive focus on recurring topics or thematic contents. While approaches centred on thematic or narrative content primarily organize discourse according to categories identified by researchers, Lexical Correspondence Analysis (LCA) identifies semantic dimensions emerging from patterns of lexical co-occurrence. It is therefore particularly suited to examining the relational organization of naturally occurring discourse through patterns of lexical co-occurrence, rather than through thematic categories defined a priori.

The present study applies this approach longitudinally to an oncology support group, examining how the semantic organization of naturally occurring group discourse varies over the course of 19 sessions. The study, thus, complements existing research on illness narratives, therapeutic processes, and psychosocial outcomes by focusing on temporal variation in collective discourse. LCA was conducted across four temporal blocks, with speaker roles included as illustrative variables, and the resulting semantic configurations were interpreted alongside descriptive indicators of group climate, therapeutic alliance, and participants’ open-ended responses. Taken together, these elements provide a process-oriented account of how meanings of illness emerge, are collectively reconfigured over time within a professionally facilitated psychosocial care setting.

## 2. Materials and Methods

This study adopted a longitudinal qualitative design, complemented by descriptive indicators of group process. The primary focus was the evolving organization of meaning within naturally occurring group discourse across 19 weekly sessions of an oncology support group. Verbatim transcripts of group interactions constituted the main analytic corpus, while measures of group climate and therapeutic alliance were used to contextualize the relational environment in which discursive transformations occurred.

Consistent with a semiotic-cultural and dialogical perspective, the group interaction was considered the primary unit of analysis. Individual contributions were examined not as isolated expressions of personal experience, but as components of an evolving communicative process through which meanings of illness were collectively negotiated and transformed.

To preserve temporal sensitivity, transcripts were organized into four consecutive blocks reflecting the progression of the intervention (Block 1: sessions 1–5; Block 2: sessions 6–10; Block 3: sessions 11–15; Block 4: sessions 16–19). This temporal organization was applied consistently to textual and process data to examine changes and continuities in the semantic configurations emerging across the group process.

### 2.1. Participants and Recruitment

Participants were recruited during psychological support consultations at the hospital’s Psychology Service. During these consultations, the characteristics and aims of the group intervention were described, and eligible patients were invited to participate. No incentive was provided.

Inclusion criteria were female gender, a diagnosis of cancer, age up to 70 years, and current or recent oncological treatment. Exclusion criteria included current psychological or psychotherapeutic treatment, severe and non-stabilized psychiatric conditions (e.g., psychotic spectrum disorders or severe uncontrolled depression), cognitive impairments, conditions that could compromise regular weekly attendance (e.g., work constraints), and insufficient understanding of Italian. Screening interviews were conducted to verify eligibility and to provide information about confidentiality, voluntary participation, and the possibility of withdrawing without consequences for care.

Participants were 11 women attending a hospital-based oncology support group. Their age ranged from 43 to 70 years (M = 56.7). Participants were receiving or had recently received oncological treatments, including surgery, chemotherapy, radiotherapy, hormone therapy, and biological therapy. Diagnoses included breast cancer (*n* = 7), ovarian cancer (*n* = 2), lung cancer (*n* = 1), and head and neck cancer (*n* = 1). The group included participants at different points in their illness trajectories, with variation in diagnosis and treatment experiences, providing an interactional context in which diverse meanings of cancer could emerge and be negotiated.

Across the 19-session intervention, attendance ranged from 4 to 10 participants per session (M = 6.05, SD = 2.02). Not all participants attended every session, resulting in changes in group composition over time. Four participants discontinued participation before the end of the program, corresponding to an attrition rate of 36.4%. Discontinuation occurred after sessions 3, 4, 6, and 15 and was mainly related to clinical scheduling constraints and treatment-related fatigue, factors frequently reported as challenges in psychosocial oncology interventions (Naeyaert et al., 2026).

### 2.2. Intervention

The intervention was a structured psychological support group program grounded in a semiotic-dialogical and psychodynamic framework and aimed at supporting the elaboration of illness experience through interpersonal exchange and group meaning-making. Brief embodied practices, including tapping techniques, were integrated into the group process to facilitate attention to bodily experience, emotional regulation, and reflective engagement with illness-related meanings. These practices were not conceived as separate therapeutic components, but as experiential activities embedded within the dialogical process of the group, supporting participants in exploring and articulating embodied dimensions of their illness experience.

The group comprised 19 weekly sessions of 90 min each and was conducted within the hospital’s Psychology Service. The group was closed after Session 3 and was co-led by two trained facilitators, both clinical psychologists with more than five years of clinical experience. Each session followed a consistent structure consisting of an opening phase, a central group discussion phase, and a closing phase. The opening phase provided participants with an opportunity to share their current emotional state and reflect on experiences that had occurred during the previous week. Participants were invited to discuss difficulties, thoughts, emotions, and personal reflections related to their cancer experience, as well as any themes emerging from the preceding group session. This phase aimed to facilitate emotional expression, strengthen group cohesion, and promote continuity across sessions. The central phase focused on the exploration of emotionally salient illness-related experiences within a structured and supportive group setting. Drawing on EMDR-informed procedures (Shapiro, 2018) integrated within the broader therapeutic process, participants were guided in attending to emotionally meaningful cancer-related experiences and in exploring associated emotions, bodily sensations, cognitions, and personal meanings. This phase was intended to support emotional regulation, reflective engagement, and the reorganization of distressing experiences within broader frameworks of personal meaning and coping. The group context further encouraged recognition of shared themes across individual experiences and the development of new perspectives on illness-related emotional responses. The closing phase focused on stabilization and integration. Participants were guided through a brief Safe Place exercise (Shapiro, 2018), in which they were invited to imagine a place associated with feelings of safety, calm, and comfort, and to focus on the sensory details and positive emotions associated with this image. The exercise was used to facilitate grounding, emotional regulation, and a sense of safety before concluding the session. This was followed by a shared synthesis of the themes, emotions, and experiences that had emerged during the session, with the aim of supporting integration and continuity between sessions.

### 2.3. Data Sources and Contextual Process Measures

Data sources included verbatim transcripts of group sessions as the primary qualitative dataset, complemented by self-report measures of group process.

Verbatim transcripts of group sessions. All 19 group sessions were audio-recorded and transcribed verbatim. The resulting corpus included all communicative exchanges occurring during group meetings. Consistent with the semiotic-cultural perspective adopted in this study, transcripts were considered as representations of the evolving interactional field through which meanings of illness were collectively articulated and reconfigured.

Group process measures. To describe the relational context in which group discourse unfolded, two self-report measures of group process were administered approximately every two sessions, beginning at session 2 and including the final session: the Group Climate Questionnaire (GCQ) and the Group Working Alliance Scale (GWAS). These measures were not used as outcome indicators or to test intervention effectiveness, but as complementary descriptive information supporting the interpretation of discourse-based findings. The GCQ (MacKenzie, 1983) assesses participants’ perceptions of group climate across three dimensions: Engagement, reflecting trust, belonging, openness, and cohesion; Conflict, reflecting interpersonal tensions within the group; and Avoidance, reflecting reluctance to address difficulties during sessions. Items are rated on a 6-point Likert scale ranging from 1 (“not at all”) to 6 (“extremely”). In the present study, GCQ scores were analyzed descriptively across temporal blocks to characterize the perceived relational climate of the group. The GWAS is a group adaptation of the Working Alliance Inventory–Short Revised (WAI-SR; Hatcher & Gillaspy, 2006), based on Bordin’s (1979) tripartite model of therapeutic alliance. The scale assesses three dimensions of alliance within the group setting: Goals, referring to agreement regarding treatment goals; Tasks, referring to the perceived usefulness of group activities; and Bond, referring to trust, respect, and mutual support within the group. Items are rated on a 5-point Likert scale and aggregated by subscale. In the present study, GWAS scores were used descriptively to provide contextual information on participants’ perceptions of collaboration with the group facilitator across the intervention.

### 2.4. Data Analysis

The complete corpus of verbatim transcripts, including all communicative exchanges occurring during the 19 sessions, was analysed through Lexical Correspondence Analysis (LCA) using T-LAB 16-Plus (Lancia, 2023). LCA was selected because the aim of the study was not to identify explicit thematic categories, but to investigate the latent semantic organization underlying group discourse. By examining patterns of lexical association within the corpus, LCA allows the reconstruction of latent dimensions of meaning through which discourse is organized, as illustrated in previous semiotic-cultural studies using this approach (Salvatore et al., 2012; Venuleo et al., 2024, 2026a, 2026b).

Consistent with the semiotic-cultural and dialogical perspective adopted in this study, meanings were considered as emerging from relations among signs within interaction rather than as properties of individual speakers. Accordingly, discourse was treated not as a transparent vehicle for participants’ pre-existing inner meanings, but as the context in which meanings of illness were jointly negotiated, stabilized, and transformed over time. In line with this epistemological stance, the purpose of the analysis was not to recover stable subjective contents, but to reconstruct the evolving configurations of sense that became available within the group’s communicative field. Because facilitators actively contributed to shaping the interactional field, their interventions were considered constitutive components of the meaning-making process rather than external prompts. Accordingly, the analysis encompassed the group’s entire communicative field, including contributions from facilitators as well as from participants who later discontinued the intervention.

The basic analytical units were contextual segments generated through corpus segmentation and used as rows in the lexical matrix. Lemmatization and lexical preprocessing were performed before analysis, and only lemmas exceeding the minimum frequency threshold established for the corpus were included. By examining patterns of lexical association, LCA reconstructs the semantic dimensions through which discourse is organized, allowing the identification of configurations of meaning that characterize the corpus. LCA was applied to the text-by-lemma matrix to identify latent semantic dimensions structuring group discourse. Table 1 reports the main characteristics of the textual dataset.

Factor interpretation was based on lemmas with the highest contribution values to each pole (V-test values), together with the projection of illustrative variables within the factorial space. This procedure supported the interpretation of empirical lexical patterns within their discursive context. Factor labels were developed inductively through discussion among the authors; disagreements were discussed until consensus was reached, and representative excerpts from the corpus were selected to support the interpretation of each dimension. Given that two facilitators were also members of the research team, interpretation of the LCA results involved all authors, integrating clinical knowledge of the group process with methodological and theoretical perspectives.

The four consecutive blocks of sessions (Block 1: sessions 1–5; Block 2: sessions 6–10; Block 3: sessions 11–15; Block 4: sessions 16–19) and speaker role (facilitator vs. participants) were introduced as illustrative variables. Associations between these variables and the semantic dimensions were examined through V-test values, with higher absolute values indicating stronger alignment with the corresponding factorial dimension. Temporal variation was further explored by examining the position of session blocks within the factorial space. Given that attendance and group composition varied across sessions, the composition of the discourse corpus was not fully constant across the four temporal blocks: six participants contributed across all four blocks, while two additional participants contributed across three of the four blocks. Accordingly, comparisons across temporal blocks concerned changes in the semantic organization of collective group discourse over time, rather than within-participant longitudinal change.

Quantitative process measures (GCQ and GWAS) were analyzed descriptively, given the exploratory aim of the study, the small sample size, and attendance variability. Their function was not to test causal associations with discourse configurations, but to provide complementary information on the relational context in which semantic patterns emerged. Descriptive statistics were computed for each temporal block. When participants completed questionnaires more than once within the same block, intra-participant means were calculated before computing block-level means. Thus, the *n* reported for each block represents the number of participants contributing data to that block. Responses to the open-ended GCQ item were analyzed through thematic content analysis.

### 2.5. Reflexivity

Consistent with the semiotic-cultural and dialogical perspective guiding this study, reflexivity was considered an integral component of the research process. The researchers acknowledged that the analysis represented a situated interpretative process rather than a neutral extraction of meanings from the data.

Two members of the research team were also the facilitators who conducted the group sessions. This dual role was acknowledged as part of the researchers’ positioning within the study and was considered in the interpretation of findings. Rather than seeking to separate the researchers from the interactional field under investigation, their involvement was understood as an inherent feature of the dialogical process being studied. Accordingly, facilitators’ contributions were retained in the corpus and interpreted as part of the communicative field through which meanings of illness were produced, negotiated, and transformed.

To enhance transparency, the interpretation of semantic configurations identified through LCA was discussed within the broader research team. This collaborative process supported critical examination of interpretative assumptions and helped situate the findings within the relational and theoretical framework of the study.

## 3. Results

### 3.1. Semantic Organization of Group Discourse

Lexical Correspondence Analysis identified two main factorial dimensions, which were retained for interpretation. Because a table with 493 lexical variables produces many weakly structured dimensions, raw percentages tend to underestimate the inertia captured by the first dimensions; the Benzécri correction (Benzécri, 1979) was therefore applied. Both raw and corrected values are reported. The first dimension had an eigenvalue of 0.5065 (14.41% of raw inertia; 20.17% of corrected inertia), and the second an eigenvalue of 0.4172 (11.87% raw; 13.69% corrected). Together, the two retained dimensions accounted for 26.28% of raw inertia and 33.86% of corrected inertia. From the third dimension onwards, eigenvalues declined sharply and then flattened, with each subsequent dimension accounting for less than 10% of corrected inertia.

These dimensions should be understood as contrasting semantic configurations structuring the corpus, rather than as sequential stages of group development. The poles of each dimension represent contrasting directions of lexical association within the factorial space and are interpreted relationally, as configurations of meaning whose salience emerges through their relation to the opposite pole.

**Figure 1:**
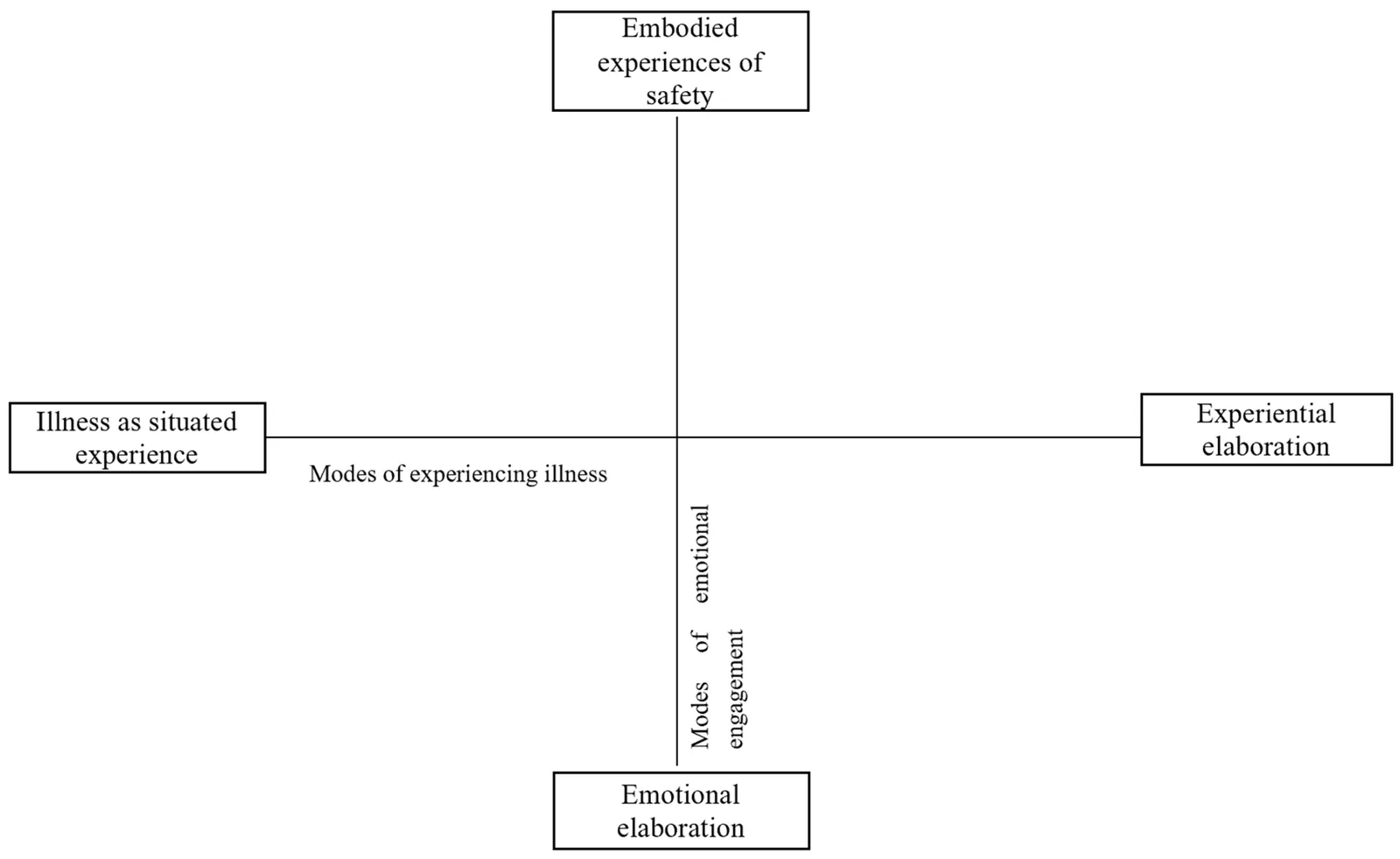
Factorial space defined by the first two dimensions of the lexical correspondence analysis. The horizontal and vertical axes represent the first and second factorial dimensions, respectively. The opposing poles of each dimension correspond to the contrasting semantic configurations identified in the corpus: Illness as situated experience versus Experiential elaboration along Factor 1, and Emotional elaboration versus Embodied experiences of safety along Factor 2.

For each factorial dimension and its corresponding poles, the lemmas with the highest levels of association (V-test values) are reported (Table 2 and Table 3; Figure 1), together with their interpretation as dimensions of semantic variability. Lemmas (italicized in the text) are translated from Italian, the language used by group members. Interpretations were grounded in representative excerpts from the corpus to support the semantic interpretation of the identified dimensions.

#### 3.1.1. First Factorial Dimension: Modes of Experiencing Illness

The first factorial dimension was characterized by two contrasting lexical configurations, which we interpreted as reflecting two modes of experiencing illness.

Illness as situated experience (−). The negative pole is characterized by lexical references to the concrete and situated dimensions of oncological illness. Lemmas refer to the situated oncological experience, encompassing its medical framing (*tumor*, *CT scan*, *problem*), the relational and familial ties involved (*son*, *mother*, *husband*, *sister*, *friend*), and interactions within clinical or shared healthcare settings (*doctor*, *people*), including communicative and interactional actions (*to call*, *to speak*, *to see*, *to take*). Additional lemmas refer to temporal markers (*day*, *month*, *year*) that structure the unfolding of events. Illustrative excerpts include:

“Even from the CT scan they wrote me an urgent referral letter, we started contacting people here and there, and we chose one of the best doctors. It went almost well, because it was already at the second stage, it was four centimeters, it was starting to enter the cerebellum.”

“He said: ‘Listen, ma’am, do you know you have a small nodule?’ He said, ‘Let’s call things by their name and surname, is it a tumor?’ He said yes, ‘didn’t you notice?’ I said, ‘notice what? Where do I have it, on the right or on the left?’”

“My son told me: ‘Mum, don’t worry, we’ll get a wig, we’ll get one with different hair, we’ll do this and that…’”

Experiential elaboration (+). The positive pole is characterized by lexical units referring to bodily awareness, sensory experience, and reflective engagement with lived experience. Co-occurring lemmas refer to bodily and sensory dimensions (*body*, *breath*, *air*, *eye*, *to close*), perceptual and experiential processes (*felt*, *sensation*, *to perceive*, *tried*), and experiential practices used within the group process (*tapping*). Additional lemmas referred to internal experiences (*memory*, *disturbance*, *disturbing*) and to states associated with well-being and safety (*well-being*, *safe*, *place*). Illustrative excerpts include:

“I notice that when we start doing tapping, at first my breathing is shorter, and then little by little it becomes longer, and this gives me a sense of peace.”

“I felt relaxed.”

“As soon as I started focusing on my breathing, I relaxed. But when you mentioned the safe place, I sank down, I really sank down. I was trying to get out; the colors were there, but they were too high up. I was in a ditch, in a well. Everything was black. I started digging with my fingernails, trying to climb out. The upper part was all colorful, like a rainbow, but I couldn’t get back up, and I couldn’t even see what was below.”

#### 3.1.2. Second Factorial Dimension: Modes of Emotional Engagement

The second factorial dimension was characterized by two contrasting lexical configurations, which we interpreted as reflecting different modes of emotional engagement.

Emotional elaboration (–). The negative pole is characterized by lexical references to emotionally salient experiences. Lemmas referring to illness-related content and recollection of difficult experiences (*illness*, *memory*, *event*, *moment*, *traumatic*, *trauma*, *painful*, *image*, *negative*) co-occur with lemmas indexing processes of exploration and discussion of emotionally salient experiences (*pathway*, *to face*, *processing*, *to think*, *work*, *emotion*, *important*, *change*). Illustrative excerpts include:

“Being part of the group helped me understand many things. I realized that many of the difficulties we face in life, beyond the cancer journey itself, depend on us—on the way we deal with them, even on the way we experience suffering.”

“I realized that the people living alongside you have the same feelings and experiences that you have in relation to the illness. For me, it was difficult to fully process that.”

“From what I am realizing, and also from what we are telling each other, it feels as if the main illness—that is, the cancer—has, at this moment, moved into the background compared to everything that is happening around it, or am I wrong? So we started out with the idea that we were supposed to, so to speak, take care of the psychological side of the illness, but instead we find ourselves dealing with, in addition to that, many other problems that have emerged.”

Embodied experiences of safety (+). In the positive polarity, co-occurring lemmas refer to natural and sensory elements (pine forest, sea, air, wind, color, green, warm, breath, smell, airflow), bodily-related terms (eye, mouth, feet), and imagery-based experiences associated with calmness, safety, and well-being (*to relax*, *place*, *home*, *peacefulness*, *well-being*, *safe)*. Illustrative excerpts include:

“The green of the trees, and if there is a bit of grass… In the pine forest where I go, there’s nothing else, just trees.”

“I can hear the cicadas and the birds. Being surrounded by nature relaxes me. I see the green of the trees, the grass, just trees. I feel well there, if I’m left alone. If I close my eyes, I can even fall asleep and relax.”

“I am in the car with my family. I go for a drive, just me and my children, toward the sea. The wind in my hair and the sound of the waves are with me, and I let myself be soothed and calmed by them.”

Figure 1 illustrates the symbolic space defined by the two dimensions of semantic variability.

#### 3.1.3. Distribution of Semantic Configurations Across Intervention Phases and Speaker Roles

The two semantic dimensions show systematic variation across temporal phases of the intervention and across speaker roles. Table 4 and Table 5 report the illustrative variables with the highest test values for each polarity.

Temporal phases. For Factor 1 (Modes of experiencing illness—Table 4), the negative polarity (Illness as situated experience) is strongly associated with intermediate and later phases of the intervention (e.g., Block 3: Meetings 11, 13–15; Block 4: Sessions 16 and 18). By contrast, the positive polarity (Experiential elaboration) reaches its clearest expression in the central phase of the group process (Block 2; e.g., Meetings 5, 7, 9, and 10), while also being detectable in some earlier sessions, particularly Meetings 2 and 3. For Factor 2 (Modes of emotional engagement—Table 5), the negative polarity (Emotional elaboration) is mainly associated with the central phase of the intervention (Block 2; sessions 6–10). At the level of individual sessions, particularly high test values are observed in Meetings 5, 6, 7, 8, 9, and 10. In contrast, the positive polarity (Embodied experiences of safety) is predominantly associated with the final phase of the intervention (Block 4; sessions 16–19), with higher test values in Meetings 11, 18, and 19, and with additional, less central associations in earlier sessions (Meetings 2 and 3).

Speaker roles. Across speaker roles, participants’ and facilitator’s discourse showed contrasting positions on both factorial dimensions. Participants’ discourse was strongly associated with the negative pole of Factor 1, Illness as situated experience, and with the positive pole of Factor 2, Embodied experiences of safety. Conversely, facilitator discourse was strongly associated with the positive pole of Factor 1, Experiential elaboration, and with the negative pole of Factor 2, Emotional elaboration.

### 3.2. Relational Context of the Intervention: Group Climate and Therapeutic Alliance

Descriptive analyses of group process measures provided contextual information on the relational environment in which the semantic configurations identified through LCA emerged.

Descriptive statistics from the Group Climate Questionnaire (GCQ) suggested a generally positive group climate across the four temporal blocks (Table 6). Engagement remained relatively high throughout the intervention, increasing from Block 1 to Block 3 and showing a slight reduction in Block 4, while still remaining comparatively elevated. Avoidance showed moderate values, with an increase in the intermediate blocks and a decrease in the final phase. Conflict scores remained low overall, despite modest fluctuations across blocks.

The Group Working Alliance Scale (GWAS) indicated overall positive perceptions of alliance and showed a descriptive pattern of higher scores in later blocks. Mean scores for Goals, Tasks, and Bond were lowest in Block 1, increased in Blocks 2 and 3, and remained comparatively high in Block 4. While Goals and Tasks reached their highest values in the final block, Bond peaked in Block 3 and showed a slight decrease thereafter, while remaining above initial levels (see Table 6).

These descriptive process measures provided contextual information on the relational environment in which the discourse configurations emerged.

The thematic analysis of open-ended GCQ responses identified five recurrent experiential and relational patterns:

(1) *Normalization through shared illness experience*: this theme reflects the normalization of emotional responses and illness experiences through peer sharing, whereby participants perceived their reactions and experiences as common within the group context.
  “We are all in the same boat. We will help each other.”
  “I recognized myself in their words and in their descriptions.”
(2) *Emergence of group belonging and emotional safety*: this theme captures the development of belonging and emotional safety within the group, which was experienced as a protective interpersonal space.
  “Sharing personal experiences within the group gives a sense of belonging.”
  “Feeling close to the group was emotional and made me feel protected.”
(3) *Emotional resonance and affective activation*: this theme describes the emotional resonance elicited by other members’ narratives, including both empathic engagement and distress.
  “When talking about people who did not make it, I feel unwell.”
  “At times I was shaken by listening to very difficult stories.”
(4) *Emergence of coping resources and new perspectives*: this theme captures the development of coping resources and new perspectives on illness experiences facilitated by group interaction. Participants described the group as a space that provided support and useful strategies for managing emotional and practical difficulties.
  “It gave me a tool to face difficult moments.”
  “This made me realize that I should try to think about my situation in a more positive way.”
(5) *Perceived safety and supportive role of group facilitators*: this theme highlights the active role of group facilitators in containing emotional experiences and fostering an environment that enabled emotional expression and personal disclosure. Facilitators were perceived not only as providing a sense of safety, but also as actively supporting participants in accessing and articulating difficult emotions and experiences.
  “The kindness of the doctor creates a safe place.”
  “Thanks to the facilitators who manage to bring out what we have inside.”

Taken together, these themes described the group as a context characterized by sharing, emotional containment, and the development of coping resources.

## 4. Discussion

This exploratory study investigated meaning-making processes within an oncology support group by examining the evolving organization of group discourse across 19 sessions. Rather than conceptualizing meaning-making as an individual achievement involving the reconstruction of a coherent illness narrative, the study approached it as a relational and dialogical process through which meanings are configured and reconfigured within interaction. Lexical Correspondence Analysis identified two main semantic dimensions organizing the group discourse: (1) a dimension contrasting illness as a situated experience with experiential elaboration, and (2) a dimension contrasting emotional elaboration with embodied experiences of safety. Importantly, these configurations did not correspond to sequential stages of group development but represented coexisting semantic resources whose relative salience varied across phases of the intervention and across speaker roles. The findings therefore suggest that meaning-making in cancer may be better understood not as the replacement of illness-related meanings with new or more adaptive interpretations, but as a changing organization of relationships among multiple ways of experiencing, interpreting, and responding to illness.

Classical accounts of biographical disruption have emphasized how serious illness challenges previously taken-for-granted assumptions about identity, continuity, and the future (Bury, 1982). Narrative approaches have further highlighted processes of reconstruction through which individuals attempt to restore continuity and coherence following illness-related disruption (Frank, 1995; Neimeyer, 2006). The present findings extend these perspectives by showing that illness-related meanings remained salient across the group discourse, while their lexical associations and contextual positioning varied over time. Rather than being replaced by more positive or elaborative meanings, illness-related configurations appeared to become embedded within broader semantic configurations involving relational, experiential, and embodied dimensions. From this perspective, meaning-making may involve not the resolution of illness-related meanings, but their repositioning within an evolving field of relations.

The first semantic dimension highlighted two ways in which illness experience was organized within group discourse. The *illness-as-situated-experience* pole was characterized by references to medical events, family relationships, healthcare interactions, and temporal markers. This configuration represents cancer as a concrete biographical event situated within personal histories, relationships, and healthcare trajectories. One possible interpretation is that such discourse provided shared reference points through which individual illness experiences could be articulated within the group. Its stronger association with later phases of the intervention should not be interpreted as a renewed dominance of illness-center thinking. Rather, illness-related discourse remained salient while occupying a changing position within a broader semantic field that also included experiential and relational dimensions. This finding resonates with narrative approaches to illness, which emphasize how biographical disruption challenges established forms of meaning and calls for processes of narrative reorganization (Bury, 1982; Kleinman, 2020). At the same time, this interpretation should be considered alongside the observed differences in semantic positioning between participant and facilitator discourse. The temporal pattern may partly reflect topics and meanings introduced or structured by facilitators, rather than exclusively spontaneous changes in participants’ illness meanings.

Conversely, the *experiential elaboration* pole was characterized by bodily awareness, sensory experience, reflective attention, and references to experiential practices. This configuration foregrounds how illness-related experiences are lived and felt, alongside their representation as concrete biographical events. This emphasis on the experiential and embodied dimensions of illness is consistent with recent qualitative research highlighting the lived body as a central dimension of cancer experience (Ahn & Lee, 2023; Sebri et al., 2022). The association of this pole with facilitator discourse and the intermediate phase of the intervention may be consistent with the role of facilitators in directing attention toward internal states, bodily experience, and reflective exploration.

The second semantic dimension further indicated that engagement with emotionally salient illness experiences was organized through different, partially complementary modes. The *emotional elaboration* pole was characterized by references to painful memories, traumatic experiences, and reflective efforts to understand emotionally salient events. This configuration foregrounds the narrative and reflective engagement with emotionally salient aspects of the illness experience. This interpretation is consistent with recent research showing that autobiographical memories and their narrative elaboration may play a role in the emotional processing and meaning-making of breast cancer experiences (Martino et al., 2023b). Similarly, group-based narrative interventions in oncology have highlighted the potential role of sharing memories and emotions in processing the traumatic experience of cancer and reflecting on its implications (Iannopollo et al., 2024). Within the present group context, the salience of this configuration may therefore indicate a discursive space in which distressing experiences could be narrated, reflected upon, and collectively re-elaborated.

The opposite pole, *embodied experiences of safety*, was characterized by sensory imagery, bodily states, natural environments, and experiences of safety and well-being. Rather than necessarily representing avoidance of difficult experiences, this configuration may reflect the availability of embodied resources through which participants could regulate emotional intensity and maintain engagement with experience. This interpretation is consistent with previous research highlighting the role of emotion regulation in psychological adjustment to cancer (Naeyaert et al., 2026). The stronger association of this configuration with later participant discourse may suggest a progressive salience of embodied and safety-related modes of experiencing across the group trajectory. Although breathing- and tapping-based activities and the Safe Place exercise were incorporated throughout the sessions, their consistent availability may have provided a stable experiential context in which participants could attend to and potentially draw upon bodily and safety-related resources over time. However, the present design does not allow us to determine whether the increasing salience of this configuration reflects an effect of the intervention, a broader process of group development, or changes in participants’ meaning-making over the course of the group.

Taken together, these two poles suggest that group-based meaning-making involves multiple and complementary resources for approaching illness-related experience. Reflective elaboration of painful experiences and embodied experiences associated with safety and well-being need not be understood as alternative stages or opposing directions; rather, they may represent different modes of engaging with emotionally salient material. From a semiotic-cultural perspective, these findings are consistent with an understanding of adaptation not as the replacement of maladaptive meanings with adaptive ones, but as an ongoing process through which multiple available meaning configurations may become increasingly differentiated and integrated, and their relationships progressively reconfigured (Evans et al., 2026; Salvatore & Freda, 2011; Venuleo et al., 2018).

The temporal distribution of semantic configurations further suggests changing relative salience rather than linear progression. The central phase of the intervention showed stronger associations with experiential and emotional elaboration, whereas later sessions showed stronger associations with situated illness discourse and embodied experiences of safety. These patterns should not be interpreted as evidence of a developmental sequence; rather, they indicate that different semantic resources became more or less prominent at different points in the group process. This view of meaning-making as a non-linear process is consistent with longitudinal research showing that sense-making in cancer unfolds through heterogeneous narrative functions and reconstructive strategies rather than through a unitary progression toward closure or resolution (Evans et al., 2026; Martino et al., 2023a). Importantly, because the unit of analysis was the group interaction, these temporal patterns refer to the configurations of meaning emerging within the specific communicative fields of each block, rather than to changes occurring within the same set of individual participants over time. At the collective level, these findings therefore concern how different configurations of meaning became more or less salient within a shared communicative field over time. Such temporal variation may also be shaped by changes in group composition and attendance, differences in participants’ contributions to the corpus, and the discourse introduced or structured by facilitators. Other contextual factors, including increasing group familiarity, specific experiential activities, and changes in participants’ clinical circumstances, may likewise have contributed to the observed variation, although their specific contribution cannot be disentangled in the present design.

The process measures provided complementary information about the relational context in which the discourse patterns unfolded. GCQ scores indicated relatively high engagement and low conflict across blocks, while GWAS scores showed generally positive perceptions of collaboration, with Goals and Tasks increasing across blocks and Bond reaching its highest level in Block 3 before a modest decline in Block 4. These descriptive patterns cannot establish causal relationships with the semantic configurations identified by LCA; rather, they indicate that the observed discursive changes unfolded within a generally supportive group context, with positive perceptions of collaboration across the intervention. These findings provide a relational context for interpreting the semantic configurations identified in the discourse, suggesting that different modes of meaning-making emerged within a group environment characterized by engagement, low conflict, and increasing perceptions of collaboration. In this sense, the study draws attention to psychosocial care settings as contexts in which social relationships and professional support intersect, providing opportunities for illness experiences to be articulated and explored.

The qualitative analysis of participants’ open-ended responses provided further insight into this relational context. Participants described the group as a setting that facilitated normalization through shared illness experiences, belonging and emotional safety, emotional resonance, and the development of new coping perspectives, consistent with evidence highlighting the potential value of peer support in cancer care (Kiemen et al., 2023). Participants also emphasized the role of facilitators in creating a protected environment in which difficult experiences could be expressed and explored. These accounts complement the LCA findings by showing how participants themselves experienced the interpersonal functions of the group, particularly the interplay between peer sharing, emotional containment, and professional support.

Overall, the study suggests that oncology support groups may function as dynamic symbolic environments in which illness meanings are continuously reconfigured and interpreted rather than resolved once and for all. By examining group discourse as an evolving semantic structure, the study contributes to a process-oriented understanding of meaning-making in cancer care, highlighting how multiple ways of experiencing, narrating, and regulating illness coexist and change through social interaction. More broadly, the findings suggest that psychological adaptation to cancer can be examined not only as an individual process, but also as something that unfolds within interpersonal and healthcare contexts that provide opportunities for sharing, reflection, emotional regulation, and the development of new perspectives on illness.

### Limitations and Future Directions of Research

Several limitations should be acknowledged. First, the study involved a relatively small sample of participants (*N* = 11). While this is consistent with the exploratory and process-oriented nature of the study, it limits the transferability of these findings beyond the specific group context examined. Accordingly, the semantic dimensions identified should be understood as context-specific configurations emerging within this particular group intervention rather than as universal features of group-based meaning-making in cancer care.

Second, participants were recruited from a single hospital-based psychology service. Organizational culture, institutional context, facilitation style, and local clinical practices may have influenced the communicative dynamics observed. Further studies across different clinical settings, healthcare organizations, and intervention models are needed to examine whether similar semantic configurations emerge across contexts.

Third, the attrition rate (36.4%) should be considered when interpreting the findings. Although participant retention represents a well-documented challenge in psycho-oncology interventions, with adherence and discontinuation rates showing substantial variability across psychosocial interventions (Brunet et al., 2024), unequal participation and changes in group composition across sessions may have contributed to longitudinal variation in the lexical-semantic configurations. Nevertheless, there was substantial continuity in group composition across the four temporal blocks, with six participants contributing throughout and two additional participants contributing across three of the four blocks. Thus, the observed longitudinal patterns should be understood as reflecting the evolving organization of group discourse within a partially changing participant composition, rather than as evidence of changes occurring within the same set of individual participants over time.

Finally, the study focused on the organization of meaning-making processes within group discourse rather than on intervention effectiveness. As no control condition or standardized pre–post clinical outcome measures were included, conclusions regarding the impact of the intervention on psychological adjustment, symptom reduction, or quality of life cannot be drawn. The findings should therefore be interpreted as describing the organization and transformation of collective meaning-making processes. The overall positive reports on the GCQ and GWAS provide a preliminary indication of participants’ positive perceptions of the group experience, although these measures cannot be taken as evidence of intervention effectiveness. Future research should integrate process-oriented analyses with standardized clinical outcome measures within larger controlled designs, extending this work across multiple clinical settings, larger and more heterogeneous samples, and comparative group designs. Such studies could examine how changes in the semantic organization of group discourse relate to psychological adjustment and other therapeutic outcomes.

More broadly, the exploratory nature of the study suggests that the semantic dimensions identified are best understood as heuristic models of the organization of meaning rather than fixed categories of participants’ experiences. Rather than classifying participants or stages of adaptation, these semantic dimensions provide an interpretative framework for examining how meanings are negotiated and reconfigured through group interaction.

Integrating Lexical Correspondence Analysis with micro-process conversation analysis or sequential interaction coding may further clarify how meanings are negotiated and transformed through moment-to-moment interaction. Larger comparative studies could also investigate how changes in the semantic organization of group discourse relate to psychological adjustment and other therapeutic outcomes.

## 5. Conclusions

This study provides an exploratory process-oriented account of meaning-making within a single oncology support group by examining the evolving semantic organization of group discourse. Rather than depicting adaptation as a linear movement from illness-related disruption toward resolution, the findings suggest that, within this group, coexisting semantic configurations varied in relative salience across the intervention. Illness-related meanings did not disappear over time but remained present within broader experiential, relational, and embodied configurations.

By combining Lexical Correspondence Analysis with complementary indicators of group process, the study illustrates how discourse analysis can contribute to understanding not only what participants talk about, but also how meanings are collectively negotiated and reconfigured over time. More specifically, the findings suggest that, in the present group context, meanings of illness were not simply expressed or resolved but actively reworked through interaction. These findings therefore highlight the potential value of examining meaning-making as a process of ongoing reorganization rather than as movement toward a fixed adaptive endpoint, while making no claims about therapeutic effectiveness.

Although exploratory and context-specific, these findings are consistent with a semiotic-cultural and dialogical perspective that foregrounds meaning-making as an interactional, embodied, and temporally unfolding and refolding process. In this sense, the study contributes to process-oriented research in psycho-oncology by offering a way of examining how illness meanings are negotiated within a shared support-group context, rather than treating them solely as individual narrative outcomes.

## Figures and Tables

**Table 1:** Dataset Characteristics.

Texts in the corpus	3523
Elementary contexts	5232
Elementary contexts retained in analysis	3775 (72.15%)
Lemma selection threshold	14
Lemmas in analysis	493

Note—*Texts in the corpus*: number of interventions (individual documentary units) that make up the corpus. *Elementary contexts*: sections of text (paragraphs) characterized by the same patterns of key words. *Elementary contexts retained in analysis*: number and percentage of elementary contexts retained for the LCA. *Lemma selection threshold*: the threshold value selected to include a lemma in the analysis. *Lemmas in analysis*: number of lemmas actually included in the analysis.

**Table 2:** First factorial dimension. Modes of experiencing illness.

Illness as Situated Experience (−)		Experiential Elaboration (+)	
Lemma	Test Value *	Lemma	Test Value *
Year	−13.1464	Felt	19.0786
Son	−12.2363	Place	18.9229
Mother	−10.8467	Sensation	17.4917
Husband	−10.7179	Memory	16.7765
To call	−10.3033	Disturbance	14.7949
Tumor	−8.5498	Tried	14.6334
Doctor	−8.387	Well-being	14.1759
Home	−8.0054	To disturb	13.9744
Problem	−8.0027	Breath	13.7556
To pass	−7.9419	Tapping	13.644
Sister	−7.876	Eye	13.4594
To take	−7.8695	Body	13.3244
To see	−7.8306	To search	12.6875
Day	−7.7047	Air	12.4069
CT Scan	−7.3376	To return	12.1885
Month	−7.2706	To perceive	11.5872
People	−7.2	Safe	11.4948
To speak	−7.1711	Event	11.369
To eat	−7.0405	Attention	11.1179
Friend	−6.7817	To ask	10.431

Eigenvalue = 0.5065; raw inertia = 14.41%; Benzécri-corrected inertia = 20.17%. * Test values indicate the strength and direction of the association between each lexical or illustrative variable and the factorial dimension. Absolute test values ≥1.96 indicate statistically significant associations (*p* < 0.05, two-tailed), with larger absolute values indicating stronger associations.

**Table 3:** Second factorial dimension. Modes of emotional engagement.

Emotional Elaboration (−)		Embodied Experiences of Safety (+)	
Lemma	Test Value *	Lemma	Test Value *
Memory	−16.5964	Pine Forest	13.389
Event	−16.4796	To see	12.3593
Traumatic	−14.917	Place	12.3519
Painful	−14.1652	Air	11.8162
Moment	−12.3092	Green	11.0944
Illness	−11.4465	Eye	10.2308
Pathway	−11.2785	Wind	9.9649
To live	−10.642	Peacefulness	9.8227
To think	−10.4309	Mouth	9.7619
Important	−9.7376	To relax	9.7231
To face	−9.6479	To go out	9.4442
Change	−9.6138	Smell	9.3587
Processing	−9.5665	Breath	9.219
Trauma	−9.4705	Colour	9.0254
Live	−8.9208	Well-being	8.9816
Emotion	−8.7611	Flow	8.8928
Belief	−8.4736	Feet	8.6561
Image	−8.4178	Home	8.6042
Negative	−8.248	Safe	8.5816
Work	−8.2474	Warm	8.4557

Eigenvalue = 0.4172; raw inertia = 11.87%; Benzécri-corrected inertia = 13.69%. * Test values indicate the strength and direction of the association between each lexical or illustrative variable and the factorial dimension. Absolute test values ≥1.96 indicate statistically significant associations (*p* < 0.05, two-tailed), with larger absolute values indicating stronger associations.

**Table 4:** First Factorial Dimension (Modes of experiencing illness): associations with illustrative variables.

Illness as Situated Experience (−)		Experiential Elaboration (+)	
Illustrative Variable	Test Value *	Illustrative Variable	Test Value *
Speaker_Participant	−94.752	Speaker_Facilitator	133.3698
Meeting_11	−85.3544	Meeting_2	130.8623
Meeting_1	−65.8974	Meeting_7	65.8784
Meeting_18	−60.976	Meeting_10	62.573
Meeting_14	−43.7443	Meeting_5	61.5708
Block_3	−39.0984	Meeting_9	44.25
Meeting_15	−30.5785	Block_2	43.1786
Meeting_16	−29.3983	Meeting_3	34.5955
Meeting_13	−25.7112	Meeting_8	25.2186
Block_4	−24.4218	Meeting_6	22.7348

* Test values indicate the strength and direction of the association between illustrative variables and the factorial dimension. Absolute test values ≥1.96 indicate statistical significance (*p* < 0.05, two-tailed).

**Table 5:** Second Factorial Dimension (Modes of emotional engagement): associations with illustrative variables.

Emotional Elaboration (−)		Embodied Experiences of Safety (+)	
Illustrative Variable	Test Value *	Illustrative Variable	Test Value *
Meeting_5	−136.307	Meeting_2	183.8968
Meeting_6	−56.1588	Meeting_3	83.0904
Meeting_10	−55.672	Meeting_11	53.3929
Block_2	−50.3586	Meeting_18	43.093
Speaker_Facilitator	−48.4523	Meeting_19	39.0511
Meeting_7	−47.7475	Speaker_Participant	36.2968
Meeting_8	−47.0395	Block_4	25.8505
Meeting_9	−42.9651	Meeting_12	22.8452
Meeting_4	−17.7051	Block_1	16.3235
Meeting_15	−11.7654	Meeting_16	14.9522

* Test values indicate the strength and direction of the association between illustrative variables and the factorial dimension. Absolute test values ≥1.96 indicate statistical significance (*p* < 0.05, two-tailed).

**Table 6:** Mean scores for GCQ and GWAS by temporal block.

Measure	Block 1 (*n* = 11)	Block 2 (*n* = 8)	Block 3 (*n* = 7)	Block 4 (*n* = 7)
GCQ				
Engagement	3.91	4.28	4.78	4.37
Avoidance	3.26	3.93	3.66	3.12
Conflict	1.67	2.14	1.86	1.42
GWAS				
Tasks	3.27	3.51	3.75	3.77
Bond	3.59	3.69	4.12	3.88
Goals	3.50	3.75	4.04	4.05

Note. Block-level means were calculated by first averaging repeated questionnaire administrations within participant and block and then averaging across participants. Total scores were computed as mean item scores.

## Data Availability

Data will be made available on request.
